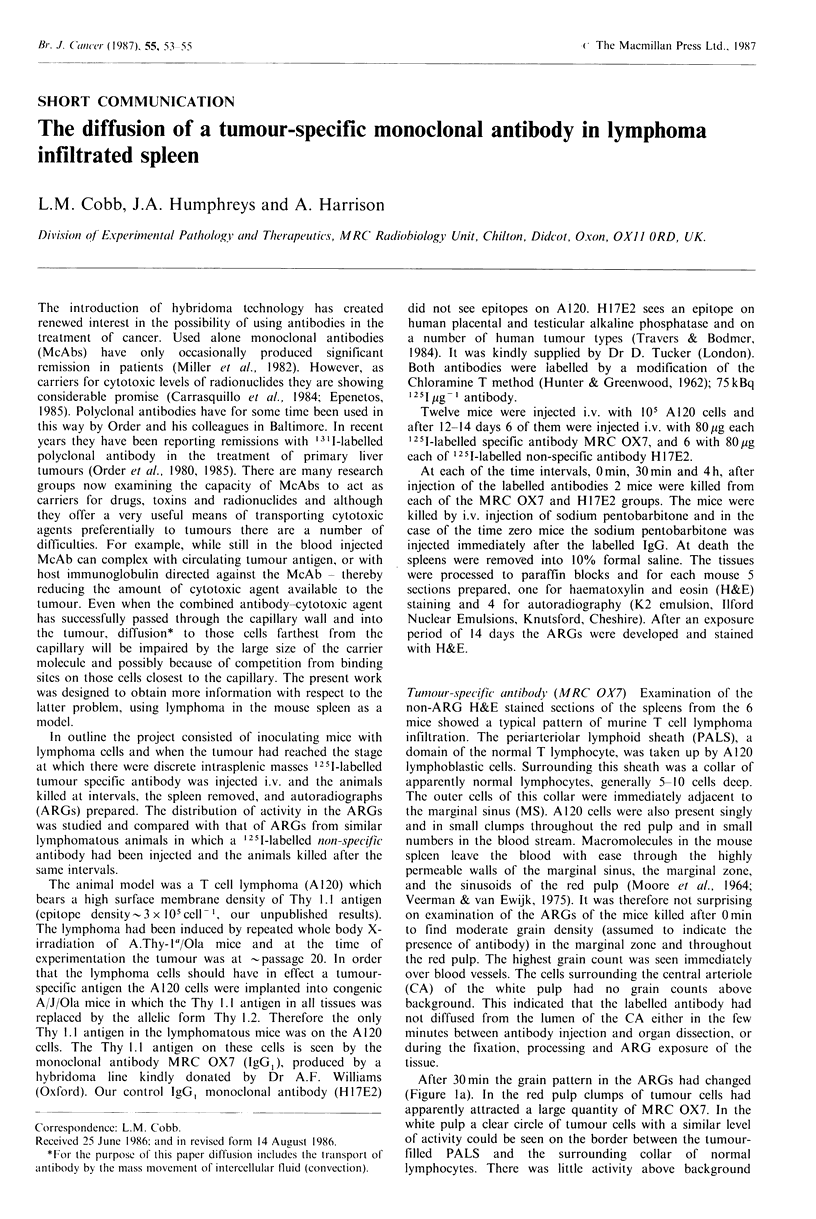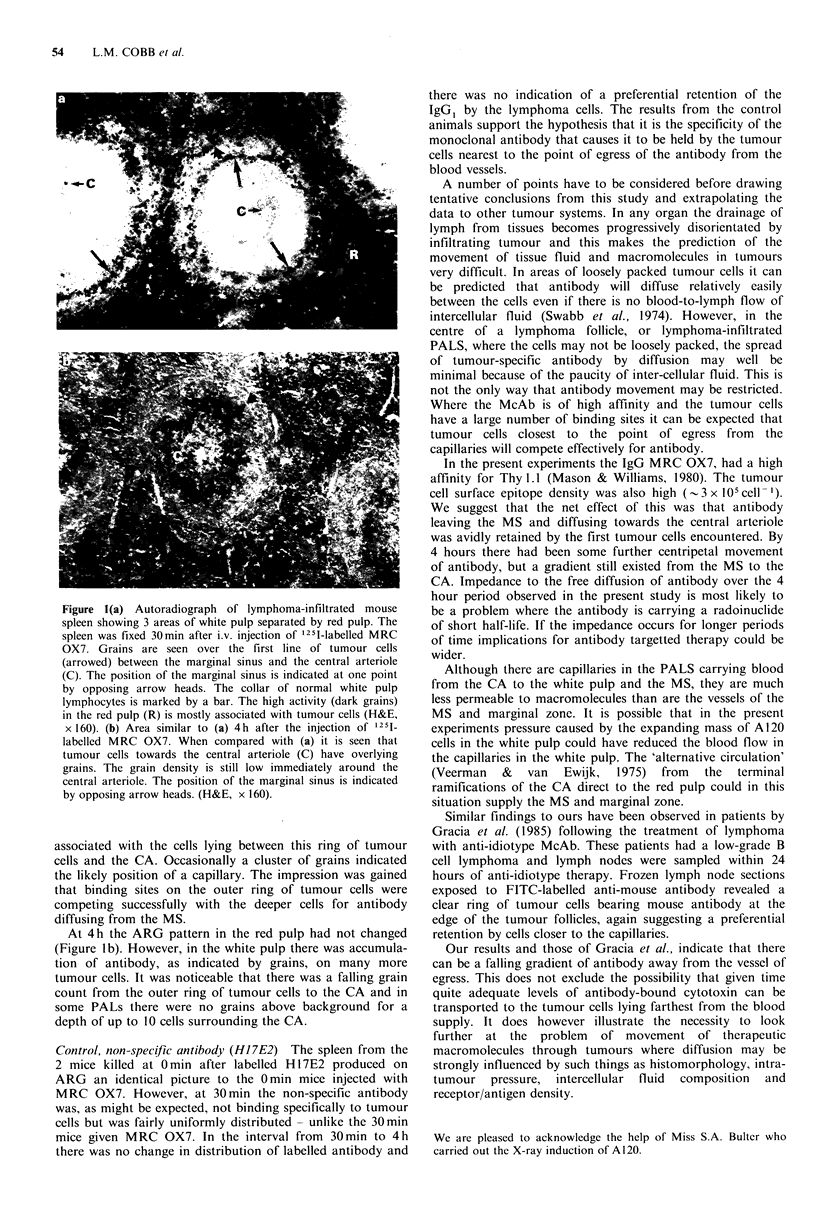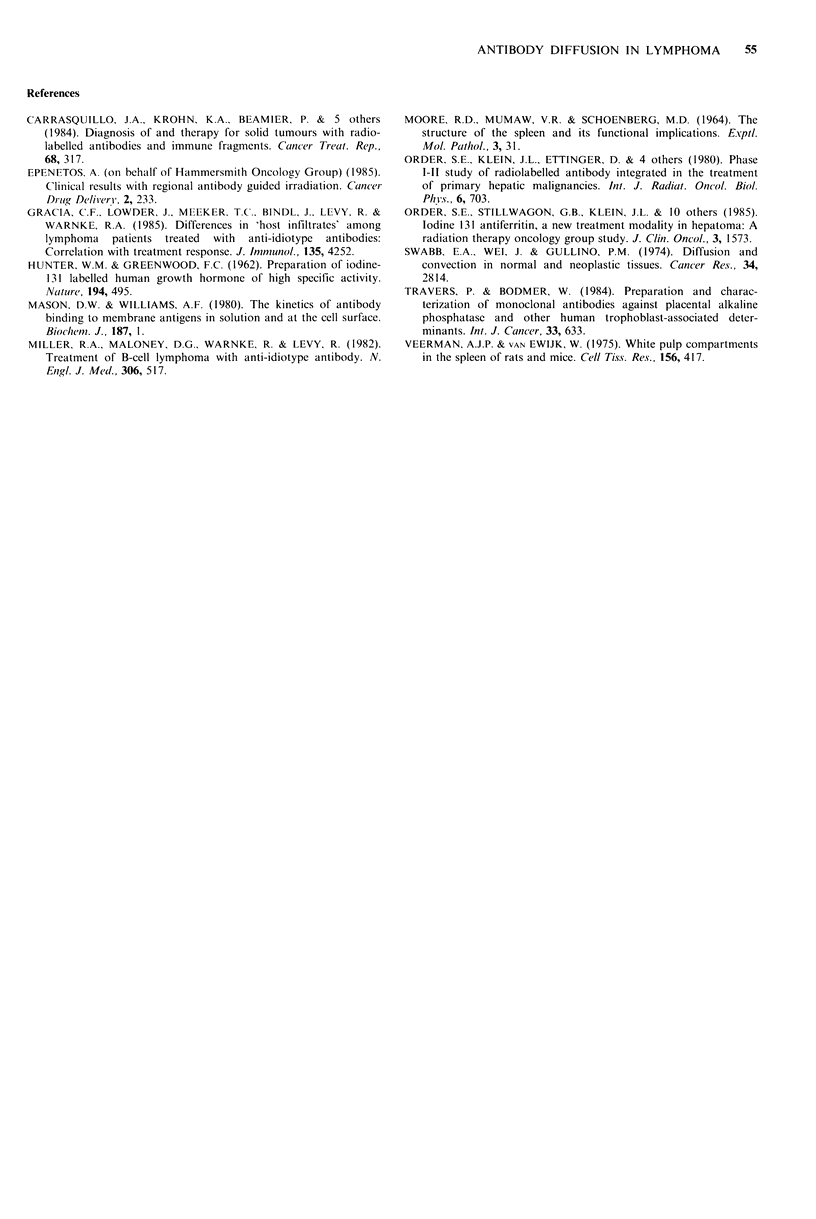# The diffusion of a tumour-specific monoclonal antibody in lymphoma infiltrated spleen.

**DOI:** 10.1038/bjc.1987.11

**Published:** 1987-01

**Authors:** L. M. Cobb, J. A. Humphreys, A. Harrison

## Abstract

**Images:**


*Br. J. Cancer* (1987), **55**, 53–55 © The Macmillan Press Ltd., 1987

SHORT COMMUNICATION

### The diffusion of a tumour-specific monoclonal antibody in lymphoma infiltrated spleen

L.M. Cobb, J.A. Humphreys and A. Harrison

*Division of Experimental Pathology and Therapeutics, MRC Radiobiology Unit, Chilton, Didcot, Oxon, OX11 0RD, UK.*

The introduction of hybridoma technology has created renewed interest in the possibility of using antibodies in the treatment of cancer. Used alone monoclonal antibodies (McAbs) have only occasionally produced significant remission in patients (Miller *et al.*, 1982). However, as carriers for cytotoxic levels of radionuclides they are showing considerable promise (Carrasquillo *et al.*, 1984; Epenetos, 1985). Polyclonal antibodies have for some time been used in this way by Order and his colleagues in Baltimore. In recent years they have been reporting remissions with $^{131}$I-labelled polyclonal antibody in the treatment of primary liver tumours (Order *et al.*, 1980, 1985). There are many research groups now examining the capacity of McAbs to act as carriers for drugs, toxins and radionuclides and although they offer a very useful means of transporting cytotoxic agents preferentially to tumours there are a number of difficulties. For example, while still in the blood injected McAb can complex with circulating tumour antigen, or with host immunoglobulin directed against the McAb – thereby reducing the amount of cytotoxic agent available to the tumour. Even when the combined antibody–cytotoxic agent has successfully passed through the capillary wall and into the tumour, diffusion* to those cells farthest from the capillary will be impaired by the large size of the carrier molecule and possibly because of competition from binding sites on those cells closest to the capillary. The present work was designed to obtain more information with respect to the latter problem, using lymphoma in the mouse spleen as a model.

In outline the project consisted of inoculating mice with lymphoma cells and when the tumour had reached the stage at which there were discrete intrasplenic masses $^{125}$I-labelled tumour specific antibody was injected i.v. and the animals killed at intervals, the spleen removed, and autoradiographs (ARGs) prepared. The distribution of activity in the ARGs was studied and compared with that of ARGs from similar lymphomatous animals in which a $^{125}$I-labelled *non-specific* antibody had been injected and the animals killed after the same intervals.

The animal model was a T cell lymphoma (A120) which bears a high surface membrane density of Thy 1.1 antigen (epitope density $\sim 3\times10^5$ cell$^{-1}$, our unpublished results). The lymphoma had been induced by repeated whole body X-irradiation of A.Thy-1$^a$/Ola mice and at the time of experimentation the tumour was at ~passage 20. In order that the lymphoma cells should have in effect a tumour-specific antigen the A120 cells were implanted into congenic A/J/Ola mice in which the Thy 1.1 antigen in all tissues was replaced by the allelic form Thy 1.2. Therefore the only Thy 1.1 antigen in the lymphomatous mice was on the A120 cells. The Thy 1.1 antigen on these cells is seen by the monoclonal antibody MRC OX7 ($IgG_1$), produced by a hybridoma line kindly donated by Dr A.F. Williams (Oxford). Our control $IgG_1$ monoclonal antibody (H17E2) did not see epitopes on A120. H17E2 sees an epitope on human placental and testicular alkaline phosphatase and on a number of human tumour types (Travers & Bodmer, 1984). It was kindly supplied by Dr D. Tucker (London). Both antibodies were labelled by a modification of the Chloramine T method (Hunter & Greenwood, 1962); 75 kBq $^{125}$I $\mu g^{-1}$ antibody.

Twelve mice were injected i.v. with $10^5$ A120 cells and after 12–14 days 6 of them were injected i.v. with 80 $\mu$g each $^{125}$I-labelled specific antibody MRC OX7, and 6 with 80 $\mu$g each of $^{125}$I-labelled non-specific antibody H17E2.

At each of the time intervals, 0 min, 30 min and 4 h, after injection of the labelled antibodies 2 mice were killed from each of the MRC OX7 and H17E2 groups. The mice were killed by i.v. injection of sodium pentobarbitone and in the case of the time zero mice the sodium pentobarbitone was injected immediately after the labelled IgG. At death the spleens were removed into 10% formal saline. The tissues were processed to paraffin blocks and for each mouse 5 sections prepared, one for haematoxylin and eosin (H&E) staining and 4 for autoradiography (K2 emulsion, Ilford Nuclear Emulsions, Knutsford, Cheshire). After an exposure period of 14 days the ARGs were developed and stained with H&E.

*Tumour-specific antibody* (*MRC OX7*) Examination of the non-ARG H&E stained sections of the spleens from the 6 mice showed a typical pattern of murine T cell lymphoma infiltration. The periarteriolar lymphoid sheath (PALS), a domain of the normal T lymphocyte, was taken up by A120 lymphoblastic cells. Surrounding this sheath was a collar of apparently normal lymphocytes, generally 5–10 cells deep. The outer cells of this collar were immediately adjacent to the marginal sinus (MS). A120 cells were also present singly and in small clumps throughout the red pulp and in small numbers in the blood stream. Macromolecules in the mouse spleen leave the blood with ease through the highly permeable walls of the marginal sinus, the marginal zone, and the sinusoids of the red pulp (Moore *et al.*, 1964; Veerman & van Ewijk, 1975). It was therefore not surprising on examination of the ARGs of the mice killed after 0 min to find moderate grain density (assumed to indicate the presence of antibody) in the marginal zone and throughout the red pulp. The highest grain count was seen immediately over blood vessels. The cells surrounding the central arteriole (CA) of the white pulp had no grain counts above background. This indicated that the labelled antibody had not diffused from the lumen of the CA either in the few minutes between antibody injection and organ dissection, or during the fixation, processing and ARG exposure of the tissue.

After 30 min the grain pattern in the ARGs had changed (Figure 1a). In the red pulp clumps of tumour cells had apparently attracted a large quantity of MRC OX7. In the white pulp a clear circle of tumour cells with a similar level of activity could be seen on the border between the tumour-filled PALS and the surrounding collar of normal lymphocytes. There was little activity above background

Correspondence: L.M. Cobb.
Received 25 June 1986; and in revised form 14 August 1986.
*For the purpose of this paper diffusion includes the transport of antibody by the mass movement of intercellular fluid (convection).

**Figure 1(a)** Autoradiograph of lymphoma-infiltrated mouse spleen showing 3 areas of white pulp separated by red pulp. The spleen was fixed 30 min after i.v. injection of $^{125}$I-labelled MRC OX7. Grains are seen over the first line of tumour cells (arrowed) between the marginal sinus and the central arteriole (C). The position of the marginal sinus is indicated at one point by opposing arrow heads. The collar of normal white pulp lymphocytes is marked by a bar. The high activity (dark grains) in the red pulp (R) is mostly associated with tumour cells (H&E, ×160). (**b**) Area similar to (**a**) 4 h after the injection of $^{125}$I-labelled MRC OX7. When compared with (**a**) it is seen that tumour cells towards the central arteriole (C) have overlying grains. The grain density is still low immediately around the central arteriole. The position of the marginal sinus is indicated by opposing arrow heads. (H&E, ×160).

associated with the cells lying between this ring of tumour cells and the CA. Occasionally a cluster of grains indicated the likely position of a capillary. The impression was gained that binding sites on the outer ring of tumour cells were competing successfully with the deeper cells for antibody diffusing from the MS.

At 4 h the ARG pattern in the red pulp had not changed (Figure 1b). However, in the white pulp there was accumulation of antibody, as indicated by grains, on many more tumour cells. It was noticeable that there was a falling grain count from the outer ring of tumour cells to the CA and in some PALs there were no grains above background for a depth of up to 10 cells surrounding the CA.

*Control, non-specific antibody* (*H17E2*) The spleen from the 2 mice killed at 0 min after labelled H17E2 produced on ARG an identical picture to the 0 min mice injected with MRC OX7. However, at 30 min the non-specific antibody was, as might be expected, not binding specifically to tumour cells but was fairly uniformly distributed – unlike the 30 min mice given MRC OX7. In the interval from 30 min to 4 h there was no change in distribution of labelled antibody and there was no indication of a preferential retention of the $IgG_1$ by the lymphoma cells. The results from the control animals support the hypothesis that it is the specificity of the monoclonal antibody that causes it to be held by the tumour cells nearest to the point of egress of the antibody from the blood vessels.

A number of points have to be considered before drawing tentative conclusions from this study and extrapolating the data to other tumour systems. In any organ the drainage of lymph from tissues becomes progressively disorientated by infiltrating tumour and this makes the prediction of the movement of tissue fluid and macromolecules in tumours very difficult. In areas of loosely packed tumour cells it can be predicted that antibody will diffuse relatively easily between the cells even if there is no blood-to-lymph flow of intercellular fluid (Swabb *et al.*, 1974). However, in the centre of a lymphoma follicle, or lymphoma-infiltrated PALS, where the cells may not be loosely packed, the spread of tumour-specific antibody by diffusion may well be minimal because of the paucity of inter-cellular fluid. This is not the only way that antibody movement may be restricted. Where the McAb is of high affinity and the tumour cells have a large number of binding sites it can be expected that tumour cells closest to the point of egress from the capillaries will compete effectively for antibody.

In the present experiments the IgG MRC OX7, had a high affinity for Thy 1.1 (Mason & Williams, 1980). The tumour cell surface epitope density was also high ($\sim 3\times10^5$ cell$^{-1}$). We suggest that the net effect of this was that antibody leaving the MS and diffusing towards the central arteriole was avidly retained by the first tumour cells encountered. By 4 hours there had been some further centripetal movement of antibody, but a gradient still existed from the MS to the CA. Impedance to the free diffusion of antibody over the 4 hour period observed in the present study is most likely to be a problem where the antibody is carrying a radioinuclide of short half-life. If the impedance occurs for longer periods of time implications for antibody targetted therapy could be wider.

Although there are capillaries in the PALS carrying blood from the CA to the white pulp and the MS, they are much less permeable to macromolecules than are the vessels of the MS and marginal zone. It is possible that in the present experiments pressure caused by the expanding mass of A120 cells in the white pulp could have reduced the blood flow in the capillaries in the white pulp. The 'alternative circulation' (Veerman & van Ewijk, 1975) from the terminal ramifications of the CA direct to the red pulp could in this situation supply the MS and marginal zone.

Similar findings to ours have been observed in patients by Gracia *et al.* (1985) following the treatment of lymphoma with anti-idiotype McAb. These patients had a low-grade B cell lymphoma and lymph nodes were sampled within 24 hours of anti-idiotype therapy. Frozen lymph node sections exposed to FITC-labelled anti-mouse antibody revealed a clear ring of tumour cells bearing mouse antibody at the edge of the tumour follicles, again suggesting a preferential retention by cells closer to the capillaries.

Our results and those of Gracia *et al.*, indicate that there can be a falling gradient of antibody away from the vessel of egress. This does not exclude the possibility that given time quite adequate levels of antibody-bound cytotoxin can be transported to the tumour cells lying farthest from the blood supply. It does however illustrate the necessity to look further at the problem of movement of therapeutic macromolecules through tumours where diffusion may be strongly influenced by such things as histomorphology, intratumour pressure, intercellular fluid composition and receptor/antigen density.

We are pleased to acknowledge the help of Miss S.A. Bulter who carried out the X-ray induction of A120.

### References

CARRASQUILLO, J.A., KROHN, K.A., BEAMIER, P. & 5 others (1984). Diagnosis of and therapy for solid tumours with radiolabelled antibodies and immune fragments. *Cancer Treat. Rep.*, **68,** 317.

EPENETOS, A. (on behalf of Hammersmith Oncology Group) (1985). Clinical results with regional antibody guided irradiation. *Cancer Drug Delivery*, **2,** 233.

GRACIA, C.F., LOWDER, J., MEEKER, T.C., BINDL, J., LEVY, R. & WARNKE, R.A. (1985). Differences in 'host infiltrates' among lymphoma patients treated with anti-idiotype antibodies: Correlation with treatment response. *J. Immunol.*, **135,** 4252.

HUNTER, W.M. & GREENWOOD, F.C. (1962). Preparation of iodine-131 labelled human growth hormone of high specific activity. *Nature*, **194,** 495.

MASON, D.W. & WILLIAMS, A.F. (1980). The kinetics of antibody binding to membrane antigens in solution and at the cell surface. *Biochem. J.*, **187,** 1.

MILLER, R.A., MALONEY, D.G., WARNKE, R. & LEVY, R. (1982). Treatment of B-cell lymphoma with anti-idiotype antibody. *N. Engl. J. Med.*, **306,** 517.

MOORE, R.D., MUMAW, V.R. & SCHOENBERG, M.D. (1964). The structure of the spleen and its functional implications. *Exptl. Mol. Pathol.*, **3,** 31.

ORDER, S.E., KLEIN, J.L., ETTINGER, D. & 4 others (1980). Phase I-II study of radiolabelled antibody integrated in the treatment of primary hepatic malignancies. *Int. J. Radiat. Oncol. Biol. Phys.*, **6,** 703.

ORDER, S.E., STILLWAGON, G.B., KLEIN, J.L. & 10 others (1985). Iodine 131 antiferritin, a new treatment modality in hepatoma: A radiation therapy oncology group study. *J. Clin. Oncol.*, **3,** 1573.

SWABB, E.A., WEI, J. & GULLINO, P.M. (1974). Diffusion and convection in normal and neoplastic tissues. *Cancer Res.*, **34,** 2814.

TRAVERS, P. & BODMER, W. (1984). Preparation and characterization of monoclonal antibodies against placental alkaline phosphatase and other human trophoblast-associated determinants. *Int. J. Cancer*, **33,** 633.

VEERMAN, A.J.P. & VAN EWIJK, W. (1975). White pulp compartments in the spleen of rats and mice. *Cell Tiss. Res.*, **156,** 417.